# Use of Enoxaparin to Counteract COVID-19 Infection and Reduce Thromboembolic Venous Complications: A Review of the Current Evidence

**DOI:** 10.3389/fphar.2020.579886

**Published:** 2020-09-16

**Authors:** Filippo Drago, Lucia Gozzo, Li Li, Andrea Stella, Benilde Cosmi

**Affiliations:** ^1^ Department of Biomedical and Biotechnological Sciences, University of Catania, Catania, Italy; ^2^ Department of Biomedical and Biotechnological Sciences, University Hospital of Catania, Catania, Italy; ^3^ Laboratory Hepalink, Shenzen, China; ^4^ Department of Specialty, Diagnostic and Experimental Medicine, University of Bologna, Bologna, Italy; ^5^ Division of Angiology and Blood Coagulation, Department of Specialty Diagnostic and Experimental Medicine, University of Bologna, Bologna, Italy

**Keywords:** enoxaparin, coronavirus, COVID-19, thromboembolism, induced thrombosis inflammation

## Abstract

The impact of the COVID-19 pandemic has been dramatic worldwide, with China, Italy, and now US at its epicenter. Researchers and clinicians are studying and testing different approaches in the attempt to prevent the infection and minimize its severity. Major efforts are focused on optimizing mechanical ventilation, antiviral, and supportive treatment; however, the role of heparin and low molecular weight (LMW) heparin in this setting has been largely overlooked. This review summarizes the available evidence about the role of heparan sulfate as a key entry mechanism for SARS-CoV-2; the efficacy of heparin and LMW heparin in counteracting its entry into the cell, the recent experimental findings obtained in *in vitro* studies using the LMW heparin enoxaparin Inhixa^®^, the role of heparin and LMW heparin in modulating the cytokine storm, and the evidence for the use of LMW heparin in the prevention and treatment of the thromboembolic complications of COVID-19. The available evidence suggests that LMW heparin appears as a promising tool in the treatment of COVID-19. Whether its systematic use is associated with a reduction in complications and ultimately mortality of these patients is being tested in several studies starting worldwide.

## Introduction

The spread of new coronavirus (SARS-CoV-2) has been recently declared a pandemic by the World Health Organization (WHO). Its dramatic impact is straining healthcare resources at their limit worldwide, first in China, then in western countries, with UK, Italy, and more recently US being the countries with the largest number of deaths to date. Researchers and clinicians are frantically studying and testing different approaches in the attempts to prevent the infection, minimize the severity, and prevent its complications (ICOTREG Group, 2020).

Four steps appear fundamental in the clinical outcome of COVID-19 infected patients: First, the cell infection by the virus; second, the so-called cytokine storm, i.e., the inflammatory response triggered by the infection; third, the pulmonary infiltration leading to a significant reduction in oxygen saturation; and lastly, the thromboembolic complications of the inflammatory response, contributing to rapid deterioration of the clinical status and death. Moreover, data are emerging indicating that diffuse bilateral pulmonary inflammation observed in COVID-19 is associated with a novel pulmonary-specific vasculopathy, which has been termed pulmonary intravascular coagulopathy as distinct to disseminated intravascular coagulation (Fogarty et al., 2020).

Mechanical ventilation and respiratory assistance remain the cornerstone treatment for patients with severe respiratory distress leading to death, especially among the elderly. On top of that, three main approaches can be envisioned to minimize the clinical consequences of COVID-19 infection: (a) prevention of virus entry into the cell and/or its replication, (b) modulation of the cytokine storm by anti-immune agents, and (c) prevention of the thromboembolic complications.

Clinical evidence about the efficacy of currently used pharmacological treatments remains scanty. At present, protocols developed in specialized centers have included the use of chloroquine and hydroxychloroquine with negative results, anti-virals such as lopinavir/ritonavir with negative results, and remdesivir, the latter with promising results and anti-inflammatory agents such as tocilizumab and desametasone with promising results. However, the search for innovative treatment approaches remains crucial for optimizing patient treatment. One overlooked research area is the attempt to inhibit the entry of SARS-CoV-2 into the cell, the very first step leading to the vicious circle described above.

This review will focus on: (a) the experimental evidence about the role of heparan sulfate as a key entry mechanism for SARS-CoV-2, (b) the efficacy of heparin and low molecular weight (LMW) heparin in counteracting its entry into the cell, (c) the recent experimental findings obtained in in-vitro studies using the LMW heparin enoxaparin (Inhixa^®^), (d) the role of heparin and LMW heparin in modulating the cytokine storm, and (e) the evidence for the use of LMW heparin in the prevention and treatment of the thromboembolic complications of COVID-19.

The available, albeit preliminary, evidence suggests that heparin in general and Inhixa^®^ in particular appear as a promising additional tool in the treatment of COVID-19.

## Heparan Sulfate as an Entry Mechanism for SARS-CoV-2

Virus tropism not only depends on its interaction with entry receptor but is also modulated by other factors, like attachment receptors, protease availability, and the activity of pathways responsible for internalization and trafficking of virus particles (Wickramasinghe et al., 2011; Promkuntod et al., 2013).

Many pathogens take advantage of the glycosaminoglycans heparan sulfate as a means to adhere and gain access to cells. Several years ago, the critical role of heparan sulfate has been clearly documented by de Haan et al. (2005). These authors have shown that murine hepatitis virus, a member of the betacoronavirus subfamily, acquires the ability to infect human cells by successive culture in infected cells thanks to the mutation, which confers the virus the ability to attach to heparan sulfate proteoglycan. Later studies confirmed that human coronavirus NL63 take advantage of heparin sulfate to attach to target cells through a structural M protein (Milewska et al., 2014; Milewska et al., 2018).

Recently, Mycroft-West C. et al. evaluated the interaction between the SARS-CoV-2 Spike S1 protein receptor binding domain (SARS-CoV-2 S1 RBD) and heparin and were able to show an interaction between the recombinant surface receptor binding domain and the polysaccharide, thus indicating the strong potential of repurposing heparin as an antiviral agent.

## Efficacy of Heparin in Counteracting the Entry of SARS-CoV-2

Heparan sulfate (HS) and heparin share similar structural characteristics, both of them are polysaccharides formed by repeated disaccharide covalently linked by uronic acid and acetylglucosamine with variable chain length and number of sulfate groups (average heparin disaccharide contains approximatively 2.7 sulfate groups, whereas heparan sulfate >1 sulfate group per disaccharide unit). In higher organisms, they can be found primarily on the cell surface or in the extracellular matrix, attached to a protein core. Heparin is a highly acidic polymer and its biological effects depend on both specific and nonspecific ionic interactions. The anticoagulant activity is related to the presence of a specific pentasaccharide sequence present in approximately 20–30% of commercially available heparin. The specific pentasaccharide sequence binds and potentiates the effect of antithrombin a naturally occurring anticoagulant, which can inhibit several serine proteases of the coagulation system, primarily FIIa (thrombin) and FXa. More recently, a heparin octasaccharidic sequence obtained by chemoenzymatic synthesis, in which glucuronic acid is replaced with sulfated iduronic acid, was shown to similarly bind to and activate antithrombin, thus paving the way for the development of heparin-like drugs that be obtained by a chemo-enzymatic approach (Elli et al., 2020).

However, heparin chains can have non anticoagulant effects by binding “nonspecifically” but also specifically to more than 100 proteins (Young, 2008). Significant clinical and basic science literature shows that heparin also possesses anti-inflammatory effects as it can modulate the function and activity of mediators of the immune response, acute phase and complement proteins, and growth factors. The activity of several proteins acting as mediators of inflammation, including CD11b/CD18, eosinophil cationic protein, IL-8, neutrophil elastase, major basic protein, P- and L-selectin, platelet growth factor 4, and stromal-derived factor 1a is modulated by heparin (Hao et al., 2019; Hippensteel et al., 2020). A direct interaction of heparin with vascular endothelial cells (ECs), reducing recruitment of the innate immune system and inhibiting neutrophil activation, has also been shown. The anti-inflammatory effects of heparin and its constituent heparan sulfate glycosaminoglycan fragments are attributable to two general mechanisms: (i) inflammation dampening through interaction with proinflammatory mediators and (ii) prevention of the adhesion and infiltration of inflammatory cells to the diseased area (Hao et al., 2019; Hippensteel et al., 2020).

However, heparin utilization as anti-inflammatory agent has been hindered by the fear of bleeding, but the pleiotropic effects of heparin and its related compounds may have greater therapeutic potential than compounds directed against a single target due to the existing connection between inflammation, atherogenesis, thrombogenesis, and cell proliferation.

A potential role of heparin in counteracting the interaction of virus with host cell has been already documented. It competes with the herpes simplex virus for host cell surface glycoproteins to limit infection (Shukla and Spear, 2001) and it prevents cell death of human neural progenitor cells induced by Zika virus (Ghezzi et al., 2017).

The possibility that the infection by a SARS-CoV strain can be inhibited by heparin was demonstrated in an experiment conducted on the sputum specimen of an Italian patient infected by SARS (Vicenzi et al., 2004). The authors documented that the virus firstly binds to the abundant HS in the extracellular matrix, increasing its density on the cell surface, and promoting the recognition to its ACE2 receptor. Heparin (100 µg/mL) added 30 min before infection of Vero cells with SARS-CoV reduced the formation of plaques by 50%.

More recent data indicated that the human coronavirus NL63 similar to SARS-CoV-2 S1 RBD undergoes conformational change upon heparin binding, and this decreases the adhesion and hence the interaction with the ACE receptors. Since the interaction with heparan sulfate acts to facilitate ACE receptors binding by virus, it is also possible to block virus cell entry by modulating ACE 2 receptors, and recently Hoffmann et al. (2020) have shown that SARS-CoV-2 cell entry is blocked by camostate mesylate, a protease inhibitor acting on ACE2 and TMPRSS2.

Mycroft-West C. J. et al. showed that the addition of heparin to Vero cells at concentration spanning therapeutic use can inhibit SARS-Cov2 invasion between 44 and 80%. Heparin and low molecular weight heparin both bind to the Spike (S1) protein receptor binding domain, inducing conformational change. A hexasaccharide is required for conformational change. These findings are implied in the process of repurposing heparin a first line therapeutic agent as an antiviral agent and tailor made GAG based antiviral agent.

Yang et al. (2020) also showed by native mass spectrometry that both short (pentasaccharide) and relatively long (eicosasaccharide) heparin oligomers form 1:1 complexes with S1 protein receptor binding domain, supporting the existence of a single binding site. This association induces a conformational change with an important reduction of the ability to associate with ACE2. Heparin destabilizing effect is greater with the longer chains because of the electrostatic repulsion between the low-pI ACE2, and the heparin segments are not accommodated on the receptor binding domain surface.

Spike protein binding and infection by SARS-CoV-2 virus is potently blocked by unfractionated heparin, non-anticoagulant heparin, treatment with heparin lyases, and purified lung heparan sulfate (Clausen et al., 2020).

Thus, the available evidence indicates that heparan sulfate has a central role in the adhesion of the virus to the cell surface and that heparin leads to a conformational change of the SARS-CoV-2 surface protein and therefore limits its interaction with the ACE2 receptor, thus inhibiting SARS-CoV-2 infection (Kim et al., 2020). Heparan sulfate manipulation or the inhibition of viral adhesion by exogenous heparin can constitute new therapeutic opportunities (Kim et al., 2020).

## Role of Enoxaparin in Modulating the Cytokine Storm

There is strong evidence indicating that a cytokine storm occurs during the evolution of SARS-CoV-2 infection. The development of cytokine storm leads ultimately to the necrosis of epithelial cells, increased permeability of vascular cells, and abnormal cellular and humoral immunity, eventually resulting in acute lung injury, acute respiratory distress syndrome (ARDS), and death (Arabi et al., 2017).

Evidence obtained in Chinese patients points to IL-6 release as a main trigger (Wan et al.,; Chen et al., 2020). In the study by Wan et al. on 123 patients, increased levels of IL-6 were observed in 76.2% of the patients with severe disease (16 of 21) compared with 30.4% of the patients with mild disease (31 of 102). Similar results were obtained in the 29 patients studied by Chen et al. In both studies, other cytokines including IL-1β, IL-8, IL-10, TNF-α, and hs-CRP were not significantly different in patients with mild vs. severe disease.

Several studies documented a role of heparin in modulating IL-6 release based on the initial observation of the heparin-binding properties of IL-6 (Mummery and Rider, 2000). For example, *in vitro* experiments demonstrated that the production of IL-6 and IL-8 induced by LPS is inhibited by heparin in human EC (Li et al., 2015) and by the non-anticoagulant fraction of enoxaparin in trypsin-treated pulmonary epithelial cells (Shastri et al., 2015).

Studies *in vivo* models indicated that the production of IL-6 and TNFα from alveolar macrophages induced by LPS can be attenuated by nebulized heparin (Chimenti et al., 2017).

Clinical data on the effect of enoxaparin on IL-6 level have been already documented several years ago (Zenáhlíková et al., 2010). However, very recent evidence suggests that LMW heparin has the potential to relieve inflammation in COVID-19 patients: in a retrospective cohort study, Shi et al. demonstrated that the use of LMW heparin was associated with a higher percentage of lymphocytes and, most importantly, a significantly lower level of IL-6, suggesting a key role of LMW heparin in modulating inflammatory response (Shi et al., 2020).

Moreover, despite the mechanism underlying COVID-19 pulmonary vasculopathy is still unclear; the expression on both type II pneumocytes and vascular EC within the lungs of the ACE2 receptor exploited by COVID-19 supports the possibility of direct pulmonary EC infection, activation, and/or damage (Varga et al., 2020). Furthermore, the cytokine storm associated with COVID-19 infection will have major impacts upon thrombin generation and fibrin deposition within the lung (Zhou et al., 2020).

## Enoxaparin and Venous Thromboembolic (VTE) Complications in COVID-19 Infection

WHO’s attention has been drawn to the vascular complications that accompany COVID-19 infection when developing severe acute respiratory syndrome (SARS). In a specific section, the interim guidance recently released (WHO, 2020) recommends thromboprophylaxis with either unfractionated or low molecular weight heparin (LMWH), since, as discussed earlier in this review, acute infections are strong prothrombotic stimuli and these patients are at increased risk of venous thromboembolism (VTE). Abnormal coagulation has been reported in a multicentre retrospective study in Chinese patients hospitalized with severe disease (Tang et al., 2020) in whom elevated D-dimer >1 gr/L was associated with in-hospital death, even after multivariate adjustment for other variables. In another study (Deng et al., 2020), non-survivors had significant higher levels of D-dimer, and 71% met the clinical criteria for disseminated intravascular dissemination (DIC).

Severe and critically ill COVID patients with prolonged immobilization are inherently at high risk of VTE, and pulmonary embolism (PE) should also be considered in those with clinical deterioration with hypoxia and hemodynamic instability. However, the optimal thromboprophylaxis regimen in hospitalized patients with COVID-related illness is unknown (Driggin et al., 2020). Standard LMWH prophylaxis may be insufficient, especially in the ICU patients who are characterized by a dynamic day-to-day variation both of thromboembolic and bleeding risk. Monitoring of anti-Xa activity may be considered when LMWH is used in these patients (Duranteau et al., 2018), and yet, failure rates with standard pharmacological prophylaxis with LMWH or UFH may not be negligible (5–15%) (Boddi, 2017). Current studies will clarify the ideal regimen in the COVID-19 clinical setting. This is even more important in light of the very recent observation of a high incidence (31%) of thrombotic complications in ICU patients with COVID-19 infections (Klok et al., 2020). The authors reinforced the recommendation to “strictly apply pharmacological thrombosis prophylaxis in all COVID-19 patients admitted to the ICU, and to increase the prophylaxis towards high-prophylactic doses, even in the absence of randomized evidence”.

So far only data regarding observational retrospective studies of either LMWH or UFH for COVID-19 related illness are available, with mixed results (Hasan et al., 2020).

There are least 14 ongoing randomized clinical trials registered in ClinicalTrials.Gov, and they are all open label comparing standard prophylactic LMWH or UFH doses vs. intermediate therapeutic LMWH doses in patients hospitalized for SARS-CoV-2 in either general wards or intensive care units (Marietta et al., 2020).

The results of these studies are awaited to draw firmer conclusions on the role of heparin in SARS-CoV-2 related illness.

## Clinical Implications and Conclusions

The experimental and clinical evidence summarized in this review suggests a strong rationale for testing the use of enoxaparin in patients with COVID-19 infection. **Table 1** summarizes the potential beneficial effects. Whether a systematic use of this treatment is associated with a reduction in complications and ultimately mortality of these patients will be defined when the results of several studies starting worldwide will be available. Although randomized clinical trials remain the ideal setting to evaluate safety and efficacy of novel treatments, the threat posed by COVID-19 requires that clinicians are able to collect data in real-world setting.

**Table 1:** Potential effects of enoxaparin in the COVID-19 infection setting.

• Prevention of infection by decreasing virus cell entry and hence viral load • Reduction of IL-6 release associated with cytokine storm • Prevention of activation of coagulation cascade • Prevention of venous thromboembolism • Prevention and treatment of thrombosis of small and middle size vessels leading to lung failure

In that respect, the fact that LWM heparin is already recommended as a preventive measure of venous thromboembolism allows clinicians to collect clinical data in real-world and help answering this crucial question for the optimal management of COVID-19 patients.

## Author Contributions

Conception/design: FD and AS. Collection and/or assembly of data: LG, LL, AS, and BC. Original draft preparation: FD, LG, LL, AS, and BC. Review and editing: LG, LL, and AS. All authors contributed to the article and approved the submitted version.

## Conflict of Interest

The authors declare that the research was conducted in the absence of any commercial or financial relationships that could be construed as a potential conflict of interest.
